# Accurate estimation of lesion metrics in radiofrequency ablation using machine learning model

**DOI:** 10.1093/ehjopen/oeag013

**Published:** 2026-02-09

**Authors:** Masateru Takigawa, Shuji Tsunoda, Takatoshi Shigeta, Junji Yamaguchi, Miho Negishi, Masaki Honda, Ryo Tateishi, Iwanari Kawamura, Tasuku Yamamoto, Kentaro Goto, Takuro Nishimura, Kazuya Yamao, Susumu Tao, Shinsuke Miyazaki, Koichi Fujiwawra, Tetsuo Sasano

**Affiliations:** Department of Cardiovascular Medicine, Institute of Science Tokyo Hospital, 1-5-45 Yushima, Bunkyo-ku, Tokyo 113-8510, Japan; Division of Advanced Arrhythmia Research, Institute of Science Tokyo Hospital, 1-5-45 Yushima, Bunkyo-ku, Tokyo 113-8510, Japan; Department of Materials Process Engineering, University of Nagoya, Chikusa-ku, Nagoya 464-8603, Japan; Department of Cardiovascular Medicine, Institute of Science Tokyo Hospital, 1-5-45 Yushima, Bunkyo-ku, Tokyo 113-8510, Japan; Department of Cardiovascular Medicine, Institute of Science Tokyo Hospital, 1-5-45 Yushima, Bunkyo-ku, Tokyo 113-8510, Japan; Department of Cardiovascular Medicine, Institute of Science Tokyo Hospital, 1-5-45 Yushima, Bunkyo-ku, Tokyo 113-8510, Japan; Department of Cardiovascular Medicine, Institute of Science Tokyo Hospital, 1-5-45 Yushima, Bunkyo-ku, Tokyo 113-8510, Japan; Department of Cardiovascular Medicine, Institute of Science Tokyo Hospital, 1-5-45 Yushima, Bunkyo-ku, Tokyo 113-8510, Japan; Department of Cardiovascular Medicine, Institute of Science Tokyo Hospital, 1-5-45 Yushima, Bunkyo-ku, Tokyo 113-8510, Japan; Department of Cardiovascular Medicine, Institute of Science Tokyo Hospital, 1-5-45 Yushima, Bunkyo-ku, Tokyo 113-8510, Japan; Division of Advanced Arrhythmia Research, Institute of Science Tokyo Hospital, 1-5-45 Yushima, Bunkyo-ku, Tokyo 113-8510, Japan; Department of Cardiovascular Medicine, Institute of Science Tokyo Hospital, 1-5-45 Yushima, Bunkyo-ku, Tokyo 113-8510, Japan; Department of Cardiovascular Medicine, Institute of Science Tokyo Hospital, 1-5-45 Yushima, Bunkyo-ku, Tokyo 113-8510, Japan; Department of Cardiovascular Medicine, Institute of Science Tokyo Hospital, 1-5-45 Yushima, Bunkyo-ku, Tokyo 113-8510, Japan; Department of Cardiovascular Medicine, Institute of Science Tokyo Hospital, 1-5-45 Yushima, Bunkyo-ku, Tokyo 113-8510, Japan; Division of Advanced Arrhythmia Research, Institute of Science Tokyo Hospital, 1-5-45 Yushima, Bunkyo-ku, Tokyo 113-8510, Japan; Division of Advanced Arrhythmia Research, Institute of Science Tokyo Hospital, 1-5-45 Yushima, Bunkyo-ku, Tokyo 113-8510, Japan; Department of Cardiovascular Medicine, Institute of Science Tokyo Hospital, 1-5-45 Yushima, Bunkyo-ku, Tokyo 113-8510, Japan

**Keywords:** Machine learning, eXtreme Gradient Boosting, Radiofrequency, Catheter ablation, Biophysics

## Abstract

**Aims:**

Conventional parameters for estimating lesions in radiofrequency (RF) catheter ablation (RFCA), such as ablation energy (AE), contact force (CF), and impedance variation, often yield suboptimal results. This study aimed to develop a machine learning (ML) model to improve the accuracy of lesion metric estimation in RFCA.

**Methods and results:**

RF energies (30–50W) were applied to excised ventricular myocardium using RFCA with CFs of 10  or 20 g for durations between 10 and 180 s, with various orientations. Correlations between total AE, force–time integral, impedance-drop, and lesion metrics were evaluated and compared to ML model predictions, using eXtreme Gradient Boosting (XGBoost). The dataset was split for training (75%) and validation (25%). Feature importance for each lesion metric was also assessed. A total of 1142 ablations were analysed. Total AE had the strongest correlation with max depth, max length, and volume (*r*^2^ = 0.63, 0.50, 0.69), followed by force–time integral (*r*^2^ = 0.54, 0.45, 0.62) and impedance-drop (*r*^2^ = 0.33, 0.45, 0.31). Impedance drop was most strongly associated with surface area (*r*^2^ = 0.48). The ML model accurately predicted lesion metrics: *r*^2^ = 0.87 for depth, 0.82 for length, 0.86 for volume, and 0.69 for surface area, with low root mean square error values. Total AE and ablation duration were key predictors, with impedance drop contributing more to surface area and length predictions.

**Conclusion:**

ML using multiple RFCA parameters improves lesion metric predictions, enhancing lesion estimation beyond conventional metrics, potentially improving procedural guidance and safety.

## Graphical Abstract

**Graphical Abstract:**
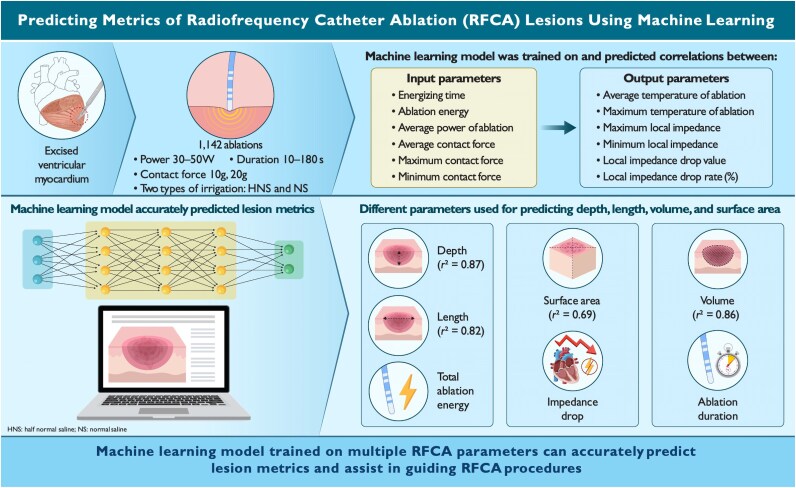

## Introduction

Radiofrequency (RF) catheter ablation (RFCA) is the standard treatment for cardiac arrhythmias,^1,2^ but procedures for atrial and ventricular arrhythmias remain suboptimal. Creating durable lesions is crucial to prevent arrhythmia recurrence.^3^ Various studies have explored input factors like RF power, contact force (CF), and duration, finding that these parameters, alone or in combination, can predict lesion size.^4–7^ Impedance is recognized as an output parameter reflecting tissue reaction during ablation and has been reported to be useful in predicting lesion size.^6,8–11^ Although combining input and output parameters has been shown to enhance predictive accuracy,^12,13^ a comprehensive model that optimally weighs these multiple variables has not been established. Furthermore, the specific contribution of each parameter to distinct lesion metrics—such as depth, length, surface area, and total volume—remains poorly understood. Therefore, this study aims to demonstrate that a machine learning (ML) model can significantly improve the predictive values of lesion metrics by integrating multiple factors. To the best of our knowledge, this represents the first attempt to utilize ML for the estimation of lesion metrics in RF ablation.

## Methods

### Experimental setup

**Figure 1:**
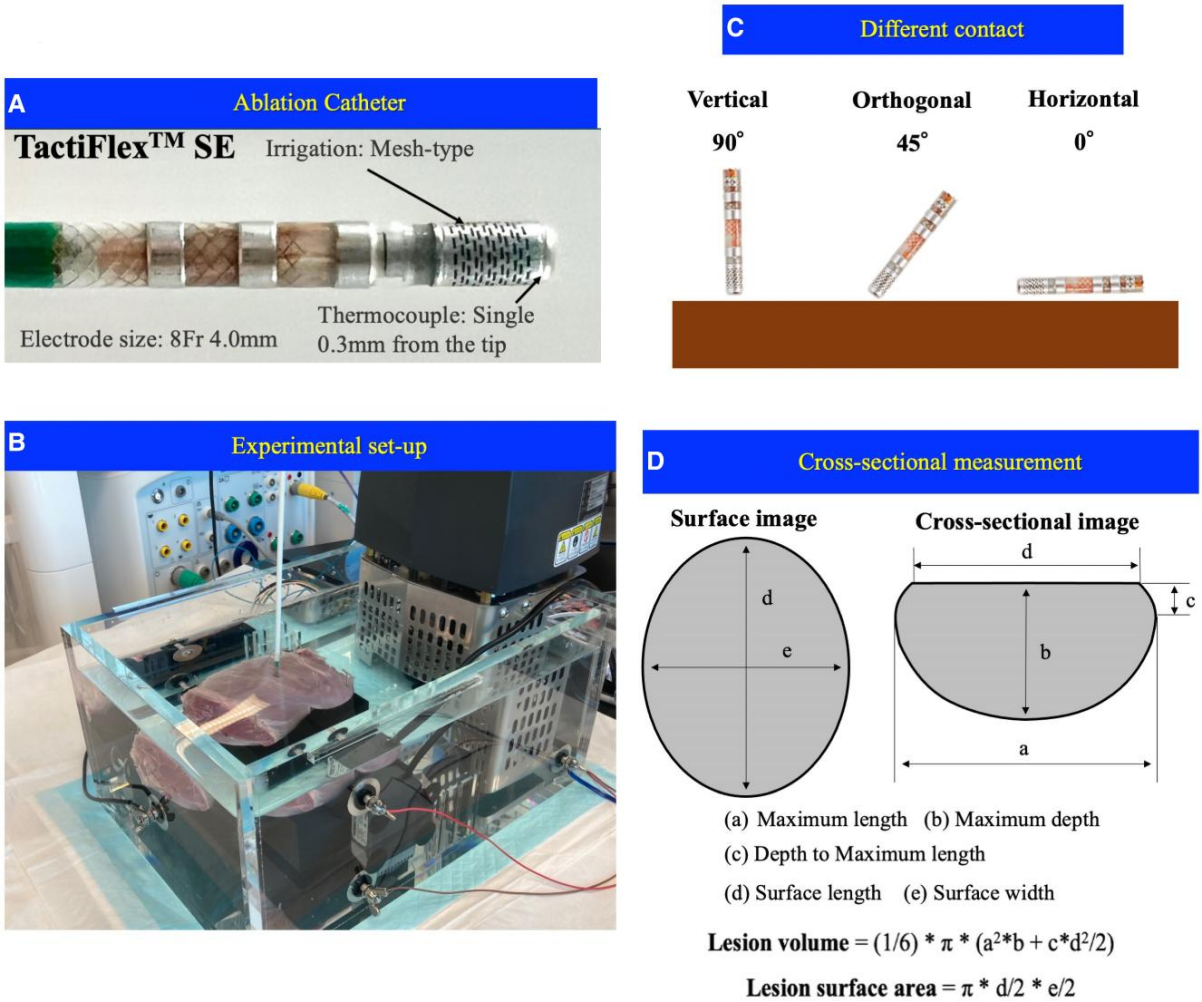
Ex-vivo experimental model. (*A*) Catheter design of the TactiFlex™ SE ablation catheter with contact sensor, installing a laser-cut 4-mm flexible 8Fr tip, which is irrigated from the proximal to distal end through the laser-cut kerfs. A single thermocouple embedded within 0.3 mm from the tip for temperature monitoring, enabling ablation with temperature control mode. (*B*) Ex-vivo experimental model. (*C*) Catheter orientations used in the experiment. Left: The ‘vertical’ (perpendicular) orientation (90°), Middle: The orthogonal orientation (45°), Right: The ‘horizontal’ (parallel) orientation (0°), as defined in the Methods.

A section of the porcine left ventricular myocardium, which was freshly excised (<12 h), was placed on the ground plate in the circulating saline bath with 5.0 L saline at 37°C. A 5 L/min flow pump was used for simulating the circulating blood flow (*Figure 1A*). To simulate the clinical setting, salinity was controlled to maintain the impedance level at 100 ± 5 Ω, measured by the catheter above the myocardial slab, based on the value in the clinical setting.

### RF applications

The TactiFlex™ SE contact-sensor ablation catheter, featuring a laser-cut 4-mm flexible 8Fr tip with a surface thermocouple positioned 0.3 mm from the tip, was employed. The catheter is irrigated from the proximal to distal end through laser-cut kerfs and from four holes on the distal end of the tip (*Figure 1A*). The Ampere™ RF generator (St Jude Medical) was connected to administer 550 kHz unmodulated sine-wave RF energy pulses in a temperature-controlled mode with a target temperature of 43°C, titrating power when the temperature approaches this level (*Figure 1B*). Various power settings, contact intensities, RF durations, and catheter orientations were employed throughout the experiment, either through a plastic pipe with or without a deflectable sheath, as detailed in the following sections.

### Lesion creation

RF energies of 30, 35, 40, 45, and 50 W were applied to the ventricular myocardium using the ablation catheter under two levels of CF (10–20 g), six durations (10, 20, 30, 60, 120, and 180 s), and three orientations relative to the tissue surface: perpendicular (vertical, 90°), parallel (horizontal, 0°), and orthogonal (45°). The catheter was irrigated with normal saline (NS, 0.9%) or half normal saline (HNS, 0.45%) at a rate of 13 mL/min (Cool Point™, Abbott) during RF application. Throughout ablation, the RF generator (Ampere™ RF Generator, Abbott) automatically recorded time-dependent ablation parameters, including RF power, duration, CF, temperature, and impedance, with a sampling rate of 0.01 s. Absolute impedance drop (ΔImp-drop) and relative impedance drop (%Imp-drop, defined as ΔImp-drop divided by the initial impedance prior to RF application) were automatically calculated and collected from the recorded data. Twelve features recorded by the system were exported for further analysis.

### Lesion size measurement

Lesions exhibiting steam pops (defined as audible pops) were excluded from the study.

The lesion border was defined as the location of a change in tissue colour. The myocardium was cross-sectioned along the surface length at the level of each lesion. Each lesion was measured with a digital calliper with a resolution of 0.1 mm by one observer who was blinded to the lesion protocol. The maximum length (a), the maximum depth (b), the depth to the maximum length (c), the surface length (d), and the surface width (e) of the created lesion were measured as shown in *Figure 1C*. The lesion surface area and the lesion volume were calculated from the following formulae:


$$\begin{array}{rl} & \mathrm{Lesion}\;\mathrm{volume}=(1/6)*\pi *(a2*b+c*d2/2),\;\\ & \;\mathrm{Lesion}\;\mathrm{surface}\;\mathrm{area}=\pi *d/2*e/2. \end{array}$$

^13,14^

### Machine learning

ML was used to construct mathematical models to estimate the lesion metrics from multiple variables recorded in a generator of the catheter. The lesion metrics estimated by the ML models in this study are as follows: the lesion volume (volume), the lesion surface area (area), the maximum depth (maxD), the maximum length (maxL), which are referred to as a Volume- model, an Area- model, a maxD- model, and a maxL- model, respectively.

Although ablation parameters were continuously recorded in a time-series sequence, the progressive growth of lesion metrics could not be measured in real time. Measuring lesion size requires interrupting the cauterization process, irreversibly excising the specimen, and then assessing its size, making continuous monitoring impractical. The ML models estimate the lesion sizes when the ablation operation finishes; however, since our experiments contained six ablation duration (10, 20, 30, 60, 120, 180 s), we can estimate the lesion sizes at these six timepoints. The model ML-model was based on the lesion metrics at these timepoints:

### Input parameters

- Duration (energizing time)
- Energy (ablation energy; AE)
- Avg Power (average power of ablation)
- Avg CF (average CF)
- Max CF (maximum CF)
- Min CF (minimum CF)

### Output parameters

- Avg Temp (average temperature of ablation)
- Max Temp (maximum temperature of ablation)
- Imp Max (maximum local impedance)
- Imp Min (minimum local impedance)
- Imp Drop (local impedance drop value) defined as Imp Max—Imp Min
- Imp Drop(%) (local impedance drop rate) defined as (Imp Max—Imp Min)/Imp Max

The ML algorithm used in this study was eXtreme Gradient Boosting^15^ (XGBoost), a fast and scalable algorithm based on gradient boosting decision trees, which enables rapid and accurate training. In addition, XGBoost can calculate variable importance that represents the degree of contribution of each explanatory variable to the estimation.

In order to train good model, hyperparameters need to be tuned appropriately. In this study, Bayesian optimization^16–18^ and cross-validation were used for hyperparameter tuning. A total of 1142 ablation records without steam-Pops were collected. The entire dataset was divided into 75% (857 records) as the training and validation dataset and 25% (285 records) as the test dataset. The training and validation dataset was used for training models and tuning their hyperparameters. A root mean square error (RMSE) of the parameter tuning dataset was used as the evaluation index of hyperparameter tuning. The test dataset was used for the evaluation of model performance. The prediction performances of the trained models were evaluated by RMSEs and the correlation coefficients.

### Statistics

The correlation between the lesion metrics and conventional parameters, including AE, impedance variation, and force–time integral, was first examined. Next, the correlation between the estimated lesion metrics using the ML model and the real lesion metrics were examined. Further, contribution of each generator variable for the prediction of each lesion metrics including lesion depth, lesion diameter, surface lesion area, and lesion volume in ML model was separately calculated. The training of the SAS screening model was conducted using Python 3.6.6 and TensorFlow 1.10.0. The significance level was set to *P* < 0.05, and computation was performed in Python 3.6.6 with SciPy 1.1.0.

## Result

### Lesion prediction using conventional parameters

**Figure 2:**
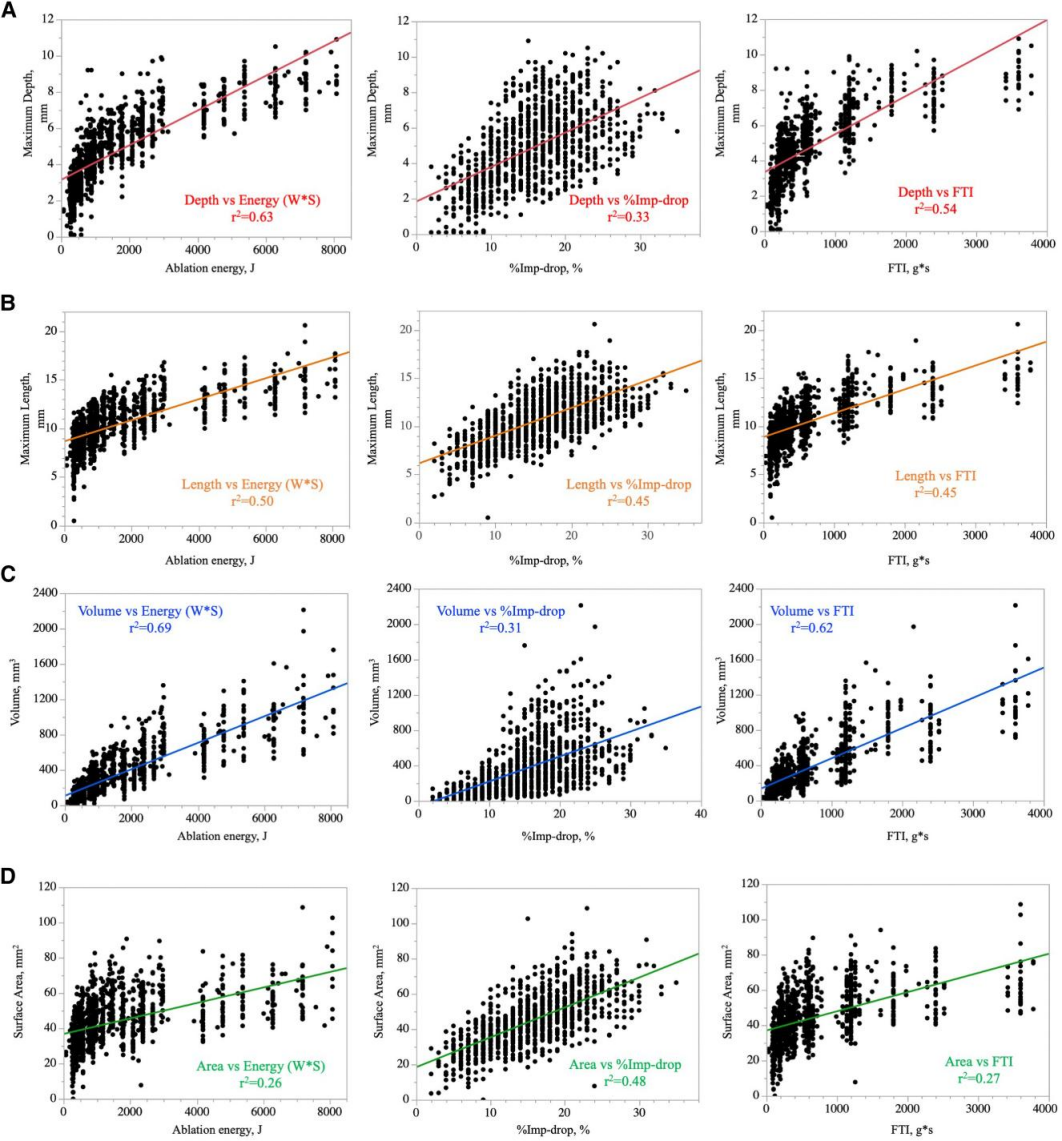
Correlation between conventional indexes and lesion metrics. Correlation between ablation energy, %impedance-drop, FTI, and (*A*) maximum lesion depth, (*B*) maximum lesion length, (*C*) lesion volume, and (*D*) lesion surface area.

Details of RF applications and the correlations between lesion metrics and conventional parameters were presented in *Table 1* and *Figure 2*. AE showed the highest correlation to the max-D, max-L, and total volume (*r*^2^ = 0.63, 0.50, and 0.69), followed by the FTI (*r*^2^ = 0.54, 0.45, and 0.62), and %impedance-drop (*r*^2^ = 0.33, 0.45, and 0.31). On the other hand, %impedance-drop showed the highest correlation to the surface area (*r*^2^ = 0.48) compared with the AE (*r*^2^ = 0.26) and FTI (*r*^2^ = 0.27).

**Table 1:** Details of radiofrequency applications

	*n* = 1142
Ave power, W	38 [30–44]
Duration, sec	30 [20–60]
Energy, J	1135 [586–2380]
Avg temperature, °C	33 [31–36]
Max temperature, °C	35 [33–39]
Max impedance, ohm	117 [110–123]
Min impedance, ohm	98 [92–106]
Impedance drop, ohm	18 [12–23]
%Imp-drop, %	16 [11–20]
Avg CF, g	16 [10–20]
Max CF, g	21 [14–24]
Min CF, g	9 [6–15]
FTI, g*sec	420 [210–1095]
Catheter direction	Vertical (*n* = 446, 39.1%)
	Orthogonal (*n* = 160, 14.0%)
	Horizontal (*n* = 536, 46.9%)
Irrigation	NS (*n* = 834, 73.0%)
	HNS (*n* = 308, 27.0%)

### Lesion prediction using ML models

**Figure 3:**
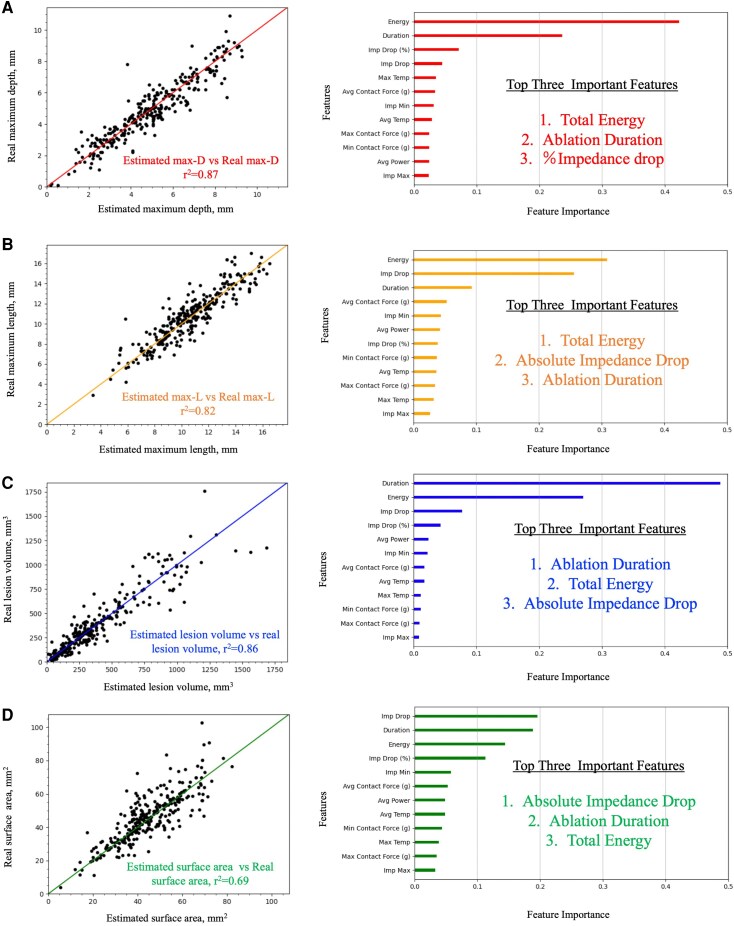
Estimation of each lesion metric and importance of specific parameters based on ML-model. Estimated maximum depth vs. real maximum depth (left panel). Ablation energy and duration are the important features followed by the %impedance drop for the estimation of lesion depth (right panel) (*A*). Estimated maximum length vs. real maximum length (left panel). Different from lesion depth, ablation duration and absolute impedance-drop are the important features followed by the duration for the estimation of lesion depth. Different from the lesion depth, ablation power also contributed to the prediction of lesion length (right panel) (*B*). Estimated lesion volume vs. real lesion volume (left panel). Ablation duration and energy are the two most important features for the estimation of lesion volume (right panel) (*C*). Estimated lesion surface area vs. real lesion surface area (left panel). Different from lesion depth, length, and volume, absolute impedance-drop is the most important feature for the estimation of the lesion surface area (right panel) (*D*).

The correlation between the estimated lesion metrics vs. the real lesion metrics, and the feature importance for predicting each metric are summarized in *Figure 3*.

### Prediction of max-D and the relevant important features

The ML-model estimated maximum lesion depth with a strong correlation (*r*^2^ = 0.87) to the real maximum depth and a small error (RMSE = 0.73 mm). In contrast, conventional parameters showed a significantly smaller correlation coefficient. AE emerged as the most important feature for predicting maximum lesion depth, followed by ablation duration and %impedance-drop. The impact of impedance variations, including both %impedance-drop and absolute impedance-drop, relatively was relatively modest, and neither ablation power nor CF contributed significantly to maximum lesion depth prediction.

### Prediction of max-L and the relevant important features

The ML-model estimated maximum lesion length with a strong correlation (*r*^2^ = 0.82) to the real maximum lesion length and a small error (RMSE = 1.09 mm). Conventional parameters exhibited a significantly smaller correlation coefficient. AE was the most important feature for predicting maximum lesion length as in the estimation of the maximum lesion depth; the second most important factor was the absolute impedance-drop. Unlike the maximum lesion depth estimation, average CF also made a minor contribution to maximum length estimation.

### Prediction of lesion volume and the relevant important features

The ML-model estimated lesion volume with a strong correlation (*r*^2^ = 0.86) to the real lesion volume and a small error (RMSE = 115 mm^3^). Conventional parameters showed a significantly smaller correlation coefficient. AE emerged as the most important feature for predicting lesion volume, followed by ablation duration and impedance variation.

### Prediction of lesion surface area and the relevant important features

The ML-model estimated lesion surface area with a strong correlation (*r*^2^ = 0.69) to the real lesion surface area and a small error (RMSE = 8.46 mm^2^). Conventional parameters exhibited a significantly smaller correlation coefficient. Compared with the estimation of the other metrics, impedance variations, including both %impedance-drop and absolute impedance-drop, are strongly contributed to lesion surface area estimation.

## Discussion

In the present study, we have demonstrated that

1. ML models based on multiple parameters including both input and output parameters can predict the lesion metrics with significantly higher accuracy compared with the conventional parameters.
2. Different important features are contributed to each lesion metric; ablation duration and total AE were important in predicting all metrics. However, the contribution of impedance variations (%impedance-drop and absolute impedance-drop) was relatively larger in predicting max-L and surface area.

### Predicting lesion metrics by ML-model

We demonstrated in the present study that the ML-model can predict specific lesion metrics based on multiple parameters, including both input and output indexes. Although some nominal variables were automatically drawn from the system and not involved in the ML model, the predictive accuracy to estimate the lesion metrics was still generally high.

RF ablation has been the mainstay of ablation therapy for approximately two decades, and the importance of transmural and durable lesions for eliminating arrhythmia origins has been well documented. Several studies have explored the variables associated with lesion expansion. Although the effect of these variables may depend on the catheter platform and ablation settings, such as power control or temperature control, parameters like power, duration, and CF are generally recognized factors associated with lesion depth, length, and volume.^4^ Subsequently, composite indices that combine multiple input parameters, such as AE, force–time integral, Ablation Index (AI), and the Lesion Size Index (LSI), have been reported to be more useful for predicting lesion size than individual parameters alone.^19,20^ It is important to note that these valuable indices are proprietary algorithms integrated into specific catheter platforms (i.e. the TactiCath and SmartTouch SF catheters, respectively). As the present study was conducted using the TactiFlex SE catheter, a direct numerical benchmark against these indices was not technically feasible. Technical feasibility aside, our ML model is fundamentally different from these approaches. The model in this study dynamically learns the complex and non-linear relationships among a much larger set of biophysical parameters (over 12 in this study). This allows for greater flexibility and adaptive weighting that fixed indices lack, potentially offering superior predictive accuracy across diverse conditions.

On the other hand, impedance variation has been reported as one of the output parameters predicting the effect of ablation and the risk of steam pops. Theoretically, impedance variation is associated with heated tissue. Since tissue temperature has been strongly correlated with lesion size, impedance variation may reflect lesion size. However, both input and output parameters and their combination are still suboptimal for predicting lesion size.

While acknowledging the predictive value of individual input and output parameters, we have recently demonstrated, in our published studies, the superiority of utilizing both types of indexes for predicting lesion metrics.^12,13^ In these investigations, we demonstrated that a simple index obtained by multiplying input parameters such as AE or force–time integral with an output parameter (%impedance-drop, defined as the ratio of absolute impedance-drop to initial impedance) significantly enhances the correlation with lesion size. Recently, ML has seen increasing utilization in the field of catheter ablation to predict and enhance clinical outcomes. Specifically, several studies have highlighted the utility of ML in predicting rhythm outcomes.^21–23^ and the progression from paroxysmal to persistent AF^24^ after AF catheter ablation.

Ablation targets such as non-PV triggers,^25^ drivers,^26^ spatio-temporal dispersion^27^ in AF have been be effectively identified by using ML algorithm. The origin of outflow tract ventricular tachycardia^28^ and the exit site of post-infarct ventricular tachycardia^29^ have been efficiently predicted by ML algorithms. However, the estimation of lesion metrics using ML, as described in the present manuscript, represents the first attempt. The results showed that multiple factors were variably associated with lesion metrics, and the ML model significantly improved the predictive values of lesion metrics.

### Feature importance

We have interestingly demonstrated that different features are associated with each lesion metric.

For the estimation of lesion depth, AE was the most important feature followed by duration. For the estimation of lesion length, AE was the most important feature followed by absolute impedance drop. To estimate the lesion volume, ablation duration was the most important feature followed by AE. While the correlation between AE, duration and the expansion of lesion size has been frequently reported,^4,7^ our present findings objectively demonstrate the individual contribution of each parameter to each lesion metrics. Although average power is a component of AE, its contribution to the estimation of lesion depth is limited, underscoring the critical role of ablation duration in determining lesion depth. Ablation power seemed to play a larger part in expanding the lesion in the horizontal direction. Impedance variation has been reported as an index to estimate lesion size,^8,9,30,31^ but its contribution is relatively lower compared to AE and ablation duration. Although impedance variation is partially associated with an increase in tissue temperature,^32^ this may not be sufficient to demonstrate the penetration of energy into deep tissue. While CF has been reported to be related to lesion size and used in combination indexes such as AI^20^ or LSI,^19^ its effect on lesion expansion is limited. Rather than CF, contact area is practically important,^10,33^ and this information may be involved in impedance variation. Therefore, CF may not play an important role in lesion estimation.

On the other hand, for predicting lesion surface area, impedance variation seemed to be strongly associated, followed by AE, with CF playing a larger role compared to the estimation of lesion volume. Since the surface lesion area should be associated with the contacted area of the heated electrode, and the impedance is associated with the catheter-tissue contact^10,33^ and tissue temperature,^32^ this finding is reasonable. It is interesting to demonstrate that lesion formation on the surface and that in the vertical direction may be associated with the different parameters for the estimation.

One of the notable findings of our feature importance analysis was the relatively low contribution of CF to lesion prediction. This may appear counter-intuitive, as CF is widely recognized as a critical parameter for lesion formation in the clinical setting.^3,4,6,20^ We believe this discrepancy is an artefact of the ex vivo model used in this study. In our controlled experimental setup, CF was maintained at stable and homogenous levels, which minimizes its variance. ML models rely on feature variance to determine predictive importance; thus, a stable parameter will naturally show a lower contribution. Conversely, in a clinical *in vivo* setting, the beating heart and respiratory motion introduce significant variability. In that dynamic environment, maintaining a consistent and adequate CF becomes a paramount factor for effective energy delivery, which is why it holds such high importance in clinical indices like the AI. Therefore, we anticipate that if our model were applied to clinical data, the feature importance of CF would increase substantially. In such a clinical environment, the influence of input and output parameters on lesion metrics would become more complex and interdependent. While the current ex vivo-trained model would likely show reduced accuracy, the inherent advantage of an ML-based approach is its capacity to learn and model these complex, non-linear interactions. We believe that by integrating dynamic data from electro-anatomic mapping systems (e.g. real-time CF logs) and qualitative data from intracardiac echocardiography (ICE) on tissue coupling, a future iteration of the model could be trained to account for this variability. This would allow for the development of a robust model that can dynamically estimate lesion metrics by adapting to the variable conditions of a real clinical procedure, a feat that is conceptually challenging for traditional fixed-formula indices.

### Clinical implication

In the present study, we have demonstrated that an XGBoost-based ML model has substantially improved lesion metric estimation in RF ablation by integrating multiple dynamic parameters throughout the ablation process, compared to estimation based on conventional ablation parameters. Moreover, it highlights the specific parameters most associated with particular lesion metrics. Applying the same methodology to in-vivo data collection will further refine the model, enhancing its relevance and applicability in clinical practice.

### Future perspective

As this study relied on an internal hold-out test set, the first step towards clinical application is to perform external validation to establish the model's robustness and generalizability. Validating its performance on a separate ex vivo dataset, ideally from a different experimental setup, is an essential next step. Following successful external validation, the logical subsequent step would be to adapt and validate the model in an *in vivo* animal model, which would incorporate the effects of perfusion and physiologic motion. Looking further ahead, the model's predictive power could be dramatically enhanced by integrating clinical data, such as catheter stability metrics from electroanatomic mapping systems and wall thickness from ICE.

In addition, the ML approach may have a potential for complication prediction. Although the present study focused on lesion size prediction, the ML methodology employed could also be applied to predict complications such as steam pops. By training a model on time-series data from ablation parameters, it may be possible to identify characteristic patterns that precede a steam pop, leading to a system that could provide real-time warnings to the operator.

### Limitations

Firstly, as our study was conducted using an ex vivo model, the results may not directly translate to those observed in an *in vivo* model or human heart. In an *in vivo* environment, new variables such as tissue contact variability, thickness of tissue, scar, etc., will mean that the output of the ML algorithm will be different and highly sensitive to these different in environment and probably operator. Secondly, the model's performance was validated using an internal dataset only; no external validation was performed. Additionally, this study did not account for scarred tissue or variations in myocardial wall thickness. However, the use of a precisely controlled model was essential to fulfil the objectives of this study, making the ex vivo model appropriate for our purposes. Consequently, the absolute values of each parameter and lesion size may be influenced by the nature of this experimental model.

## Conclusions

ML models that integrate multiple RFCA parameters offer valuable insights into the key factors influencing lesion metric predictions and enable highly accurate estimations of lesion metrics. This approach has the potential to improve the precision of RFCA procedures in clinical practice.

## Data Availability

The data underlying this article will be shared on reasonable request to the corresponding author.
